# Supplementary material for: Fermentation based carbon nanotube multifunctional bionic composites
Source: Sci Rep. 2016 Jun 9;6:27031. doi: 10.1038/srep27031 (PMC4899685; doi:10.1038/srep27031)
Supplement: Supplementary Information [file srep27031-s1.pdf]

Fermentation based carbon nanotube multifunctional bionic composites

Supporting Information

Luca Valentini,[1,*] Silvia Bittolo Bon,[1] Stefano Signetti,[2] Manoj Tripathi,[3] Erica Iacob,[3] Nicola M. Pugno[2,3,4,*]

[1] *Dipartimento di Ingegneria Civile e Ambientale, Università di Perugia, UdR INSTM, Strada di Pentima 4, I-05100 Terni - Italy.*

[2] *Laboratory of Bio-Inspired and Graphene Nanomechanics, Department of Civil, Environmental and Mechanical Engineering, University of Trento, via Mesiano 77, I-38123 Trento, Italy*

[3] *Centre for Materials and Microsystems, Fondazione Bruno Kessler, via Sommarive 18, I-38123 Povo (Trento), Italy*

[4] *School of Engineering and Materials Science, Queen Mary University of London, Mile End Road, E1 4NS London, United Kingdom.*

Corresponding authors:

Luca Valentini: Tel: +39 0744 492924; E-mail: luca.valentini@unipg.it

Nicola M. Pugno: Tel: +39 0461 282525; E-mail: nicola.pugno@unitn.it

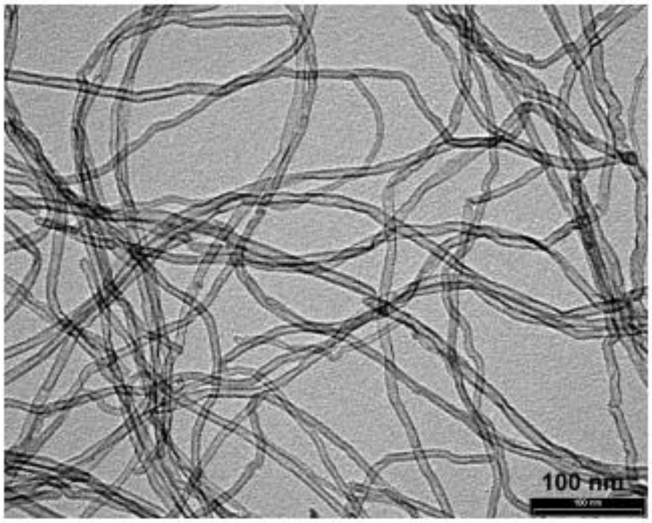

Figure S1. TEM image of multi-walled carbon nanotubes (source www.nanocyl.com).

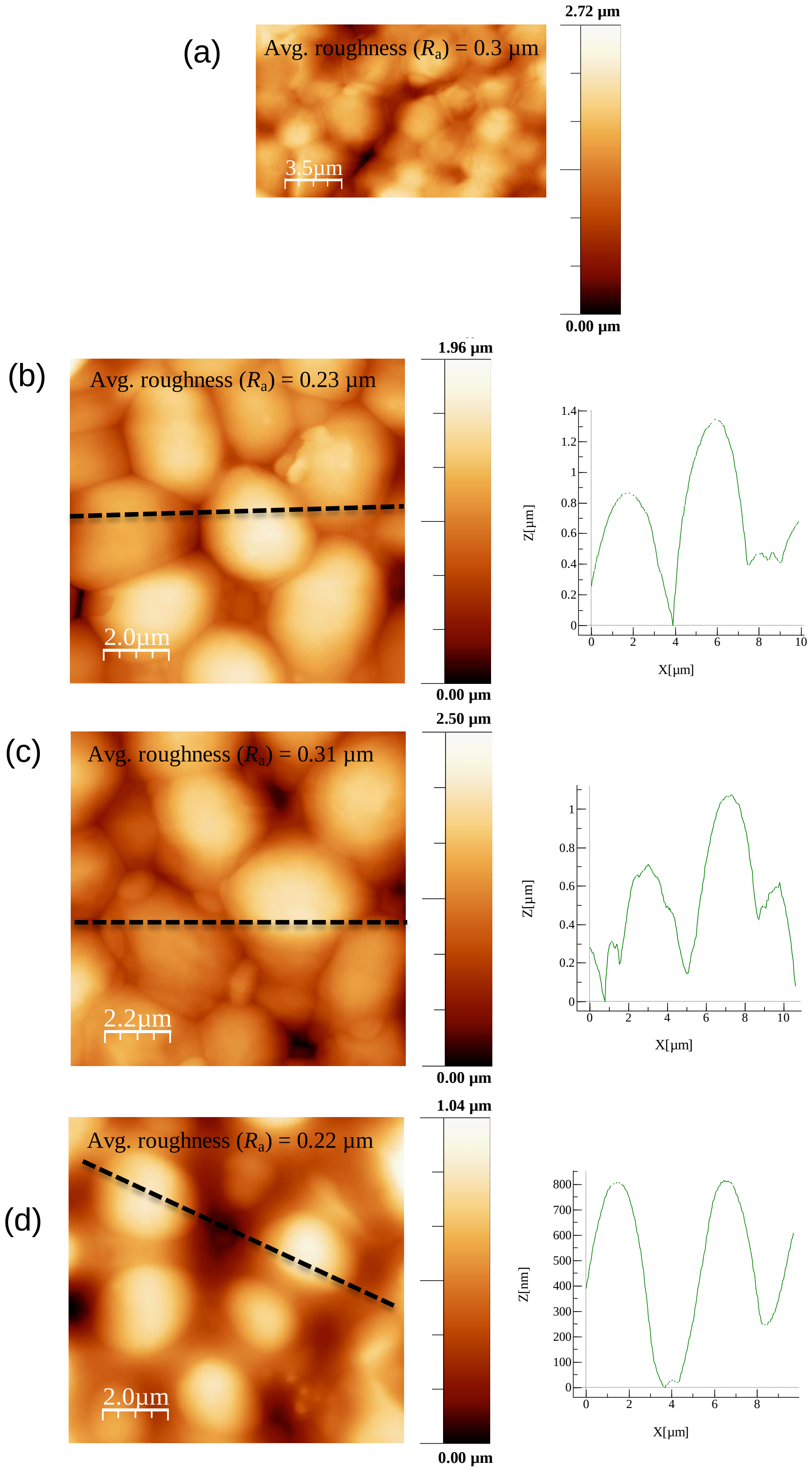

Figure S2. AFM images, average surface roughness (R_a) and surface profiles of (**a**) neat yeast, (**b**) fermented yeast, (**c**) yeast/CNTs and (**d**) fermented yeast/CNTs composite films.

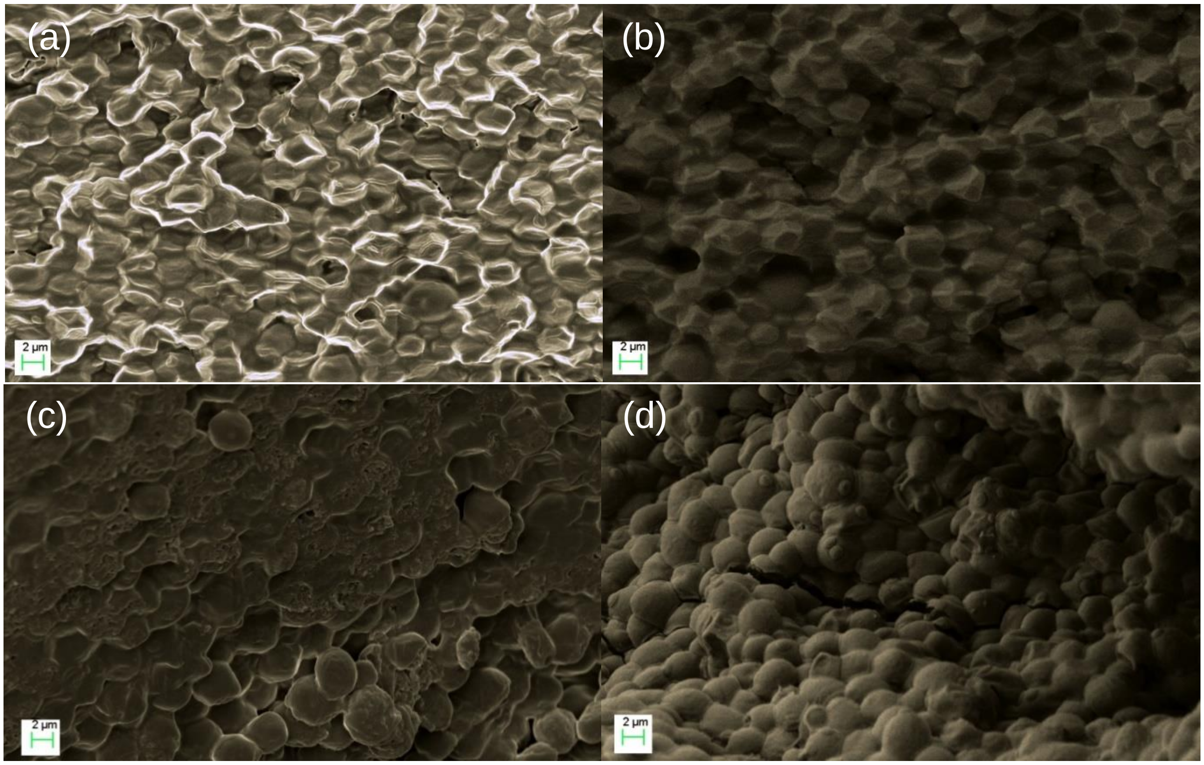

Figure S3. Cross section FESEM images of (**a**) neat yeast without fermentation, (**b**) yeast/CNTs without fermentation, (**c**) fermented yeast and (**d**) fermented yeast/CNTs films.

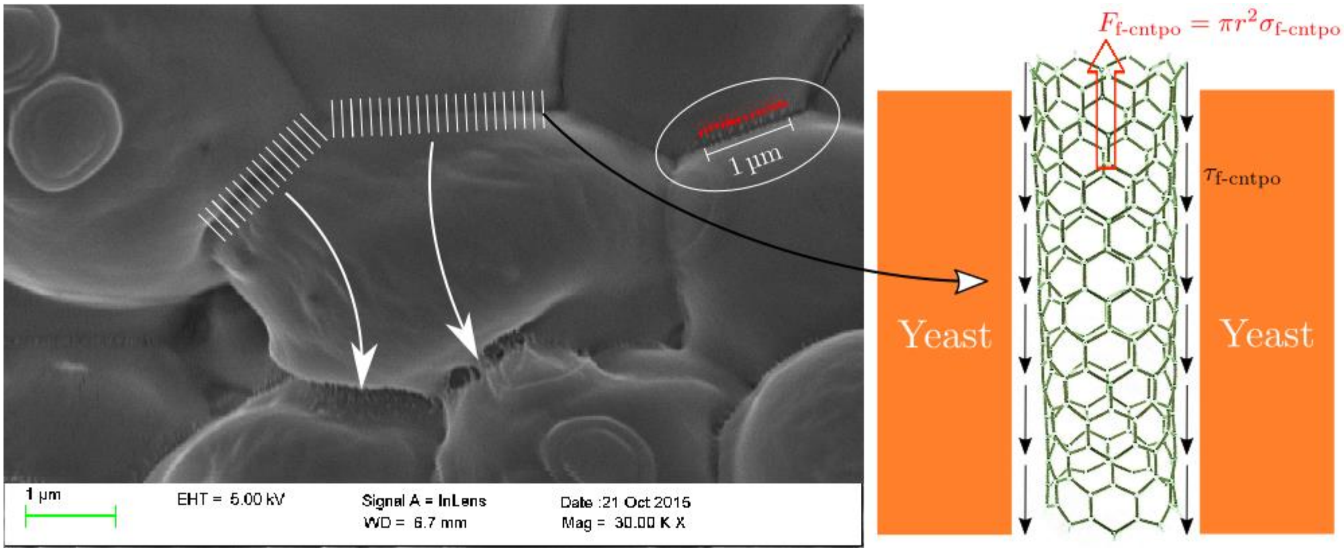

Figure S4. Schematic view of the crack bridging effect on a SEM image of the fermented yeast and pull-out mechanism. Encircled the interface at which the CNTs volume fraction was estimated by computing the number of CNTs n along a length of 1 μm (here n = 17). Assuming n^2 CNTs of 4.7 nm radius r over a 1 μm^2 interface area f is computed as areal ratio. Since CNT are multi-walled with each wall 0.34 nm thick, the total area is approximated as full section of area πr^2.

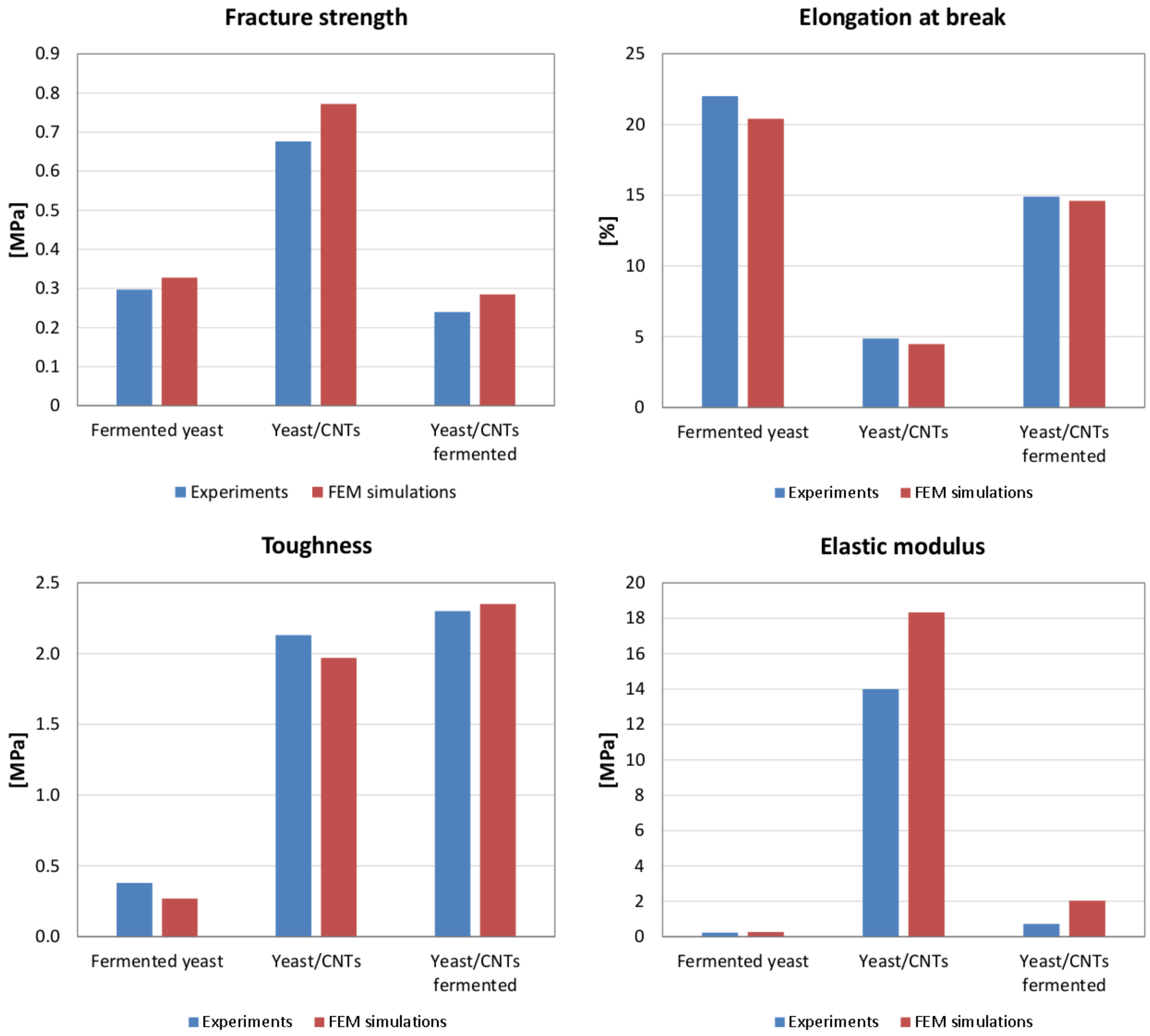

Figure S5. Characteristic parameters of the fermented yeast and of fermented yeast/CNTs composite before and after fermentation determined from the experimental constitutive curves and FEM simulations on the two-cell system (Figure 4). The fracture strength, toughness and elastic modulus of the fermented yeast sample are expressed in 10^{-1} MPa.

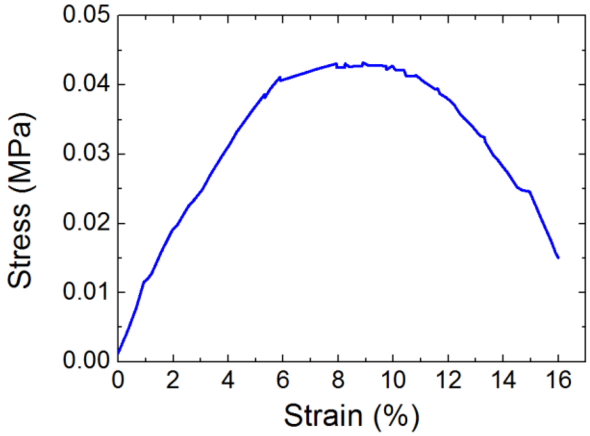

Figure S6. Stress-strain curve obtained from tensile tests on neat yeast film without fermentation.

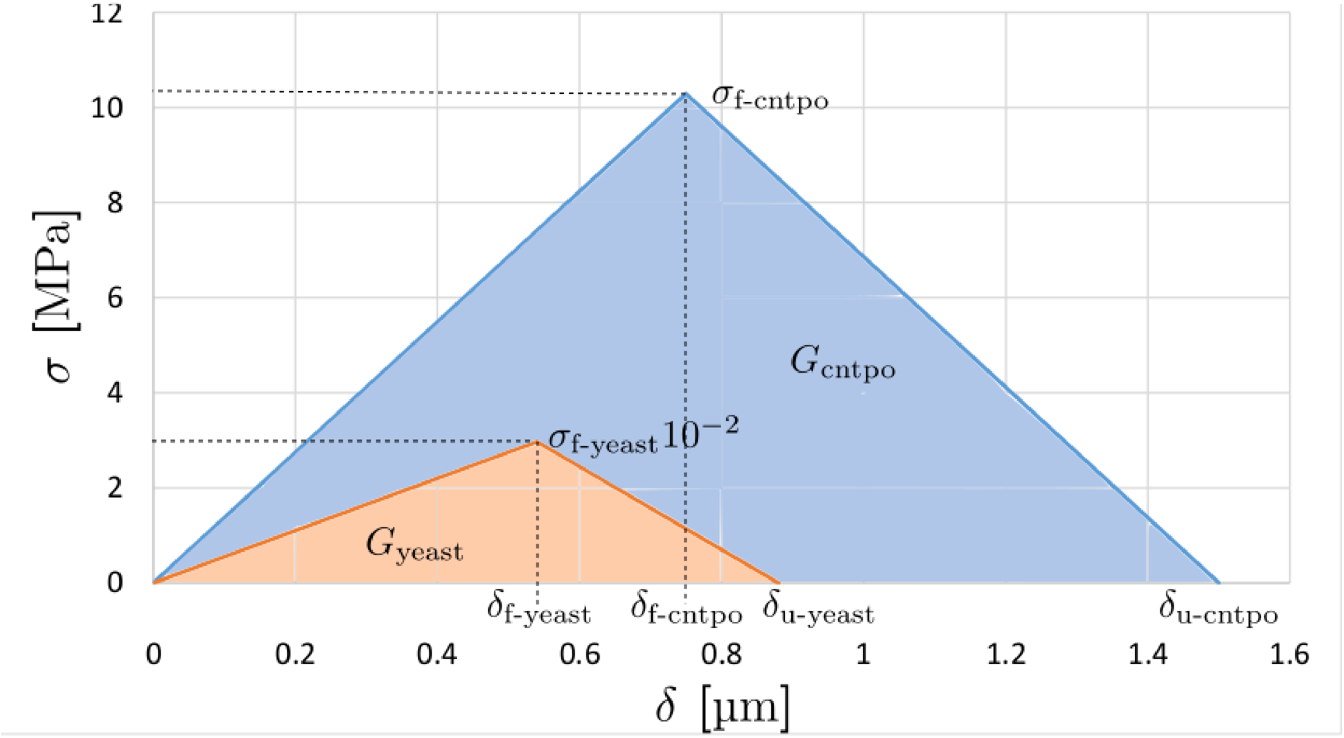

Figure S7. Cohesive laws for the yeast-yeast interface and CNTs pull out fraction

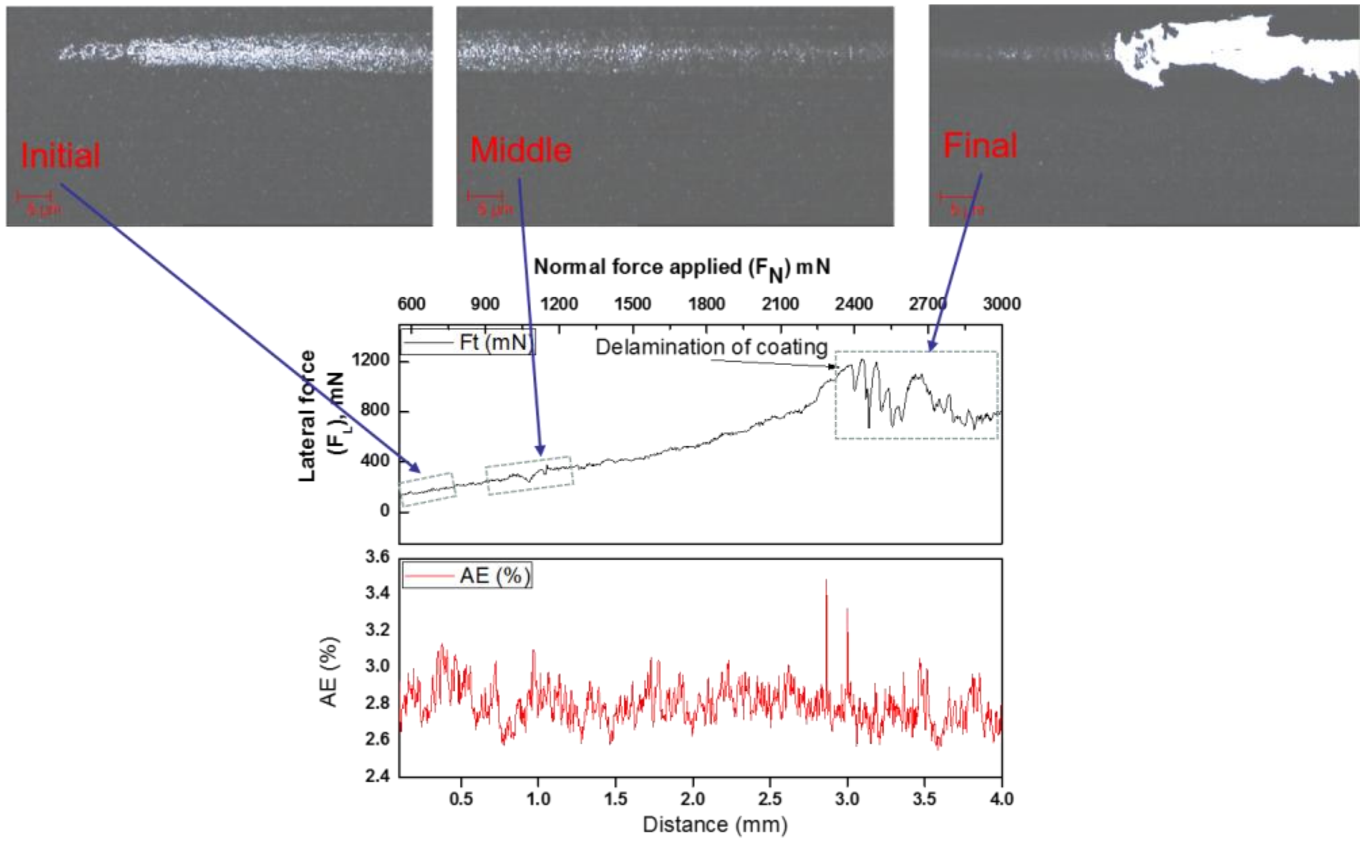

Figure S8. Lateral force, acoustic emission and delamination region of fermented yeast/CNTs composite film.
